# Chatter Stability Prediction for Deep-Cavity Turning of a Bent-Blade Cutter

**DOI:** 10.3390/s24020606

**Published:** 2024-01-18

**Authors:** Xiaojuan Wang, Qinghua Song, Zhanqiang Liu

**Affiliations:** 1 Key Laboratory of High Efficiency and Clean Mechanical Manufacture, Ministry of Education, School of Mechanical Engineering, Shandong University, Jinan 250061, China; wxjuan@mail.sdu.edu.cn (X.W.); melius@sdu.edu.cn (Z.L.); 2 National Demonstration Center for Experimental Mechanical Engineering Education, Shandong University, Jinan 250061, China

**Keywords:** deep-cavity parts, turning, bent-blade cutter, stability prediction, parameter optimization

## Abstract

The bent-blade cutter is widely used in machining typical deep-cavity parts such as turbine discs and disc shafts, but few scholars have studied the dynamics of the turning process. The existing mechanism of regenerative chatter in the metal-cutting process does not consider the influence of bending and torsional vibration, the change of tool profile and the complex machining geometry, so it cannot be directly used to reveal the underlying cause of the chatter phenomena in the deep inner cavity part turning process. This paper attempts to investigate the dynamic problem of the bent-blade cutter turning process. The dynamic model of a bent-blade cutter is proposed by considering the regenerative chatter effect. Based on the extended Timoshenko beam element (E-TBM) theory and finite element method (FEM), the coupling between the bending vibrations and the torsional vibrations, as well as the dynamic cutting forces, are modeled along the turning path. The vibration characteristics of the bending–torsion combination of cutter board and cutter bar, together with the dynamical governing equation, were analyzed theoretically. The chatter stability of a bent-blade cutter with a bending and torsion combination effect is predicted in the turning process. A series of turning experiments are carried out to verify the accuracy and efficiency of the presented model. Furthermore, the influence of cutting parameters on the cutting process is analyzed, and the results can be used to optimize the cutting parameters for suppressing machining vibration and improving machining process stability.

## 1. Introduction

**Figure 1:**
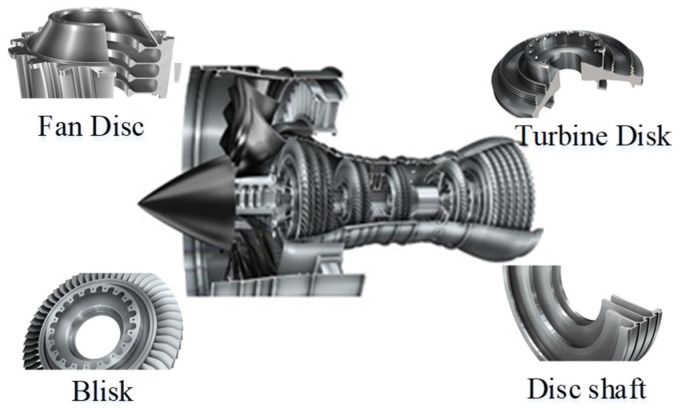
Application of deep inner cavity parts in an aeroengine.

Regarding the integrated complex deep-cavity structural parts such as the turbine disc and disc shaft in Figure 1, traditional tools can no longer meet the processing requirements, and non-standard special tools such as a bent-blade cutter need to be designed. Therefore, the prediction of machining stability is an urgent problem to be solved in deep-cavity turning of bent-blade cutters.

For weakly rigid slender parts, a smart fixture–workpiece system milling error scheme is proposed, which improves the prediction accuracy and reduces the milling error [1]. Urbikain et al. [2] developed a stability model with the Chebyshev collocation method and predicted the chatter straight turning of non-rigid parts. In the frequency domain, a linear stability analysis model for the auxiliary low-frequency vibration during the turning process has been established, along with an analytical stability prediction method for chatter in machining [3]. Khoshdarregi et al. [4] established a new threading model based on the dynamics of the thin-walled shell to research the multipoint thread-turning process of thin-walled oil pipes. Basovich et al. [5] proposed a control methodology for chatter vibration suppression with simultaneous compensation of the machining error in the internal turning process. Akdeniz et al. [6] designed the boring bar with TiNi3 alloy to reduce vibration during turning. In the process of micro-milling and orthogonal turning [7], the flexibility of the cutter is more than that of the workpiece due to a larger overhang. Therefore, the dynamic model of the tool is applied for the prediction the stability lobe diagram. In order to achieve chatter suppression, some scholars consider passive control methods such as tuned mass dampers, nonstandard cutters [8,9,10], adaptive feedback controllers [11], various damping tools [12] and additional devices [13] to enhance the stability of the machining process. All the works mentioned above focus on the turning process analysis of thin-walled cylinder parts and some vibration control strategies of the tool. For the machining of the deep inner cavity with the bent-blade cutter, there is no research yet.

The dynamic modeling of traditional metal-cutting processes, such as turning and drilling, has been deeply investigated. As the basis of dynamic characteristics analysis, modal analysis is a very important method to determine the mode of the structure. At present, establishing an accurate dynamic model is fundamental to determine, improve and optimize the dynamic characteristics of cutting tools. At the moment, the numerical, analytical and experimental are most frequently used methods. With the development of modern computer technology, the numerical method has gradually become a main method to study the dynamic characteristics of a cutting system; especially, the FEM is commonly used to establish the governing equations of the system so as to obtain the modal parameters [14,15]. Furthermore, experimental methods that are simple and obtain results easily are also widely used. Compared with the analytic method, the numerical and the experiment methods can straightforward for obtaining the static and dynamic characteristics of cutting tools. However, numerical simulations, especially for complex structures, can be computationally inefficient and challenging. Results obtained from experimental methods are more susceptible to human factors and can have larger errors. Therefore, in order to analyze the vibration response of a cutting system accurately and quickly, analytical methods have gradually become one of the main research approaches in recent years. They mainly include two aspects: one is to simplify the cutter into a lumped-parameter system [16]; the other is a continuous system [17]. The lumped-parameter system is composed of mass points connected by springs and dampers, and the parameters of the system are discrete sets with a finite element. In a continuous system, mass, elasticity and damping are distributed throughout the system. For instance, the tool can be simplified as a concentrated-parameter [18] system like the Timoshenko beam. Li et al. [19] established a flexible deformation prediction model for thin-walled components based on the principle of deformation continuity and predicted the stability of chatter vibrations. However, due to the Euler–Bernoulli beam model neglecting the influence of shear deformation and rotational inertia, its applicability is limited. Therefore, the Timoshenko beam model has become a unified and effective analytical method for studying the dynamic characteristics of cutting tools. Currently, there is a gradual consideration of the influence of tool torsional vibration in drilling and tapping processes. For instance, dynamic models for drilling and tapping processes have been established by taking into account the lateral, torsional, and axial vibrations of the cutting tool [20,21]. Abainia et al. [22] selected the geometry of turning tools from other newly designed and manufactured tools. In their experiments, they found that the choice of tool geometry is crucial. In their experiment, it was found that the selection of cutting tool geometry is greatly important. In recent years, Andreas et al. [23] have studied the influence of periodic torsional vibration of cutting tools on the dynamic modeling of metal-cutting processes. However, as mentioned above, due to the complexity of inner cavity structure, bent-blade cutter vibration is very complicated. Compared with the traditional tool, the vibration of the bent-blade cutter includes not only bending vibration, but also torsional vibration. Timoshenko beam theory cannot be directly used to model the dynamics of bent-blade cutter tools.

Regenerative chatter is one of the key factors affecting high-efficiency precision machining [24]. In order to avoid regenerative chatter, numerous studies have been conducted by researchers worldwide, including stability prediction methods, real-time monitoring, vibration control strategies. The stability lobe diagram is a commonly used method for determining the stability of the machining process, and it guides the selection of machining parameters. Mohammadi et al. [25] proposed a semi-discretization method to predict the stability of a turning system in a discrete time domain. Jiang et al. [26] proposed a full discretization method to obtain time-domain stable lobes. In order to improve the efficiency of calculation and accuracy of prediction, Li et al. [27] developed a time-varying dynamics updating method to predict the time-varying dynamics of the thin-walled parts. In addition, in the study [28,29], the influence of position-dependent and time-varying characteristics was analyzed. Compared with full discrete methods and semi-discrete methods, the DQM method is simpler and more efficient, and is widely used for solving stability differential equations [30]. Comak et al. [31] proposed a general mathematical model based on previous research. This model not only predicts the turning stability of parts but also predicts the chip thickness and cutting forces during the machining process. These methods demonstrate good stability prediction in turning, milling and drilling processes. However, due to the characteristics of the turning process for deep inner cavity parts, such as the variable tool structure, complex processing geometry and multi-directional vibrations, the stability of chatter has not been investigated in the literature.

In this study, the dynamic model of the bent-blade cutter is developed based on the turning characteristics of the cutter. The turning stability lobes of the bent-blade cutter is obtained through DQM, considering the combined bending and torsion deformation effect in the deep inner cavity part turning process. Furthermore, the influence of cutting parameters on the cutting process are analyzed, and the results can be used to optimize the cutting parameters for suppressing machining vibration and improving machining process stability. Hence, this paper is organized as follows. In Section 2, a dynamic model of the bent-blade cutter is presented, including the geometry and constitute relation of the bent-blade cutter dynamic governing equations. The finite element numerical analysis of the bent-blade cutter governing equations is in Section 3. Simulation and experimental validations are in Section 4, including the setup of the experiment, chatter tests and results analysis, and the influence of cutting parameters on turning stability. Section 5 is the conclusion of this paper.

## 2. Dynamic Model of the Bent-Blade Cutter

**Figure 2:**
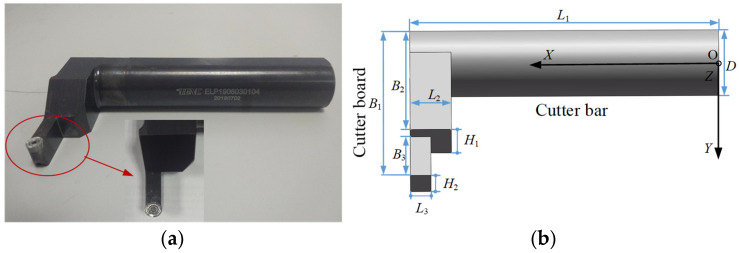
Model of bent-blade cutter (**a**) Solid model (**b**) Geometric model.

As shown in Figure 2, the complex bent-blade cutter can be regarded as a composite structure of three parts, namely the cutter bar, the cutter board I and the cutter board II. The geometric parameters of the bent-blade cutter include the total length of the bent-blade cutter (*L*_1_), the length of the cutter board I (*L*_2_), the length of the cutter board II (*L*_3_), the total width of the bent-blade cutter (*B*_1_), the width of the cutter board I (*B*_2_), the width of the cutter board II (*B*_3_), the tool diameter (*D*), the height of the cutter board I (*H*_1_) and the height of the cutter board II (*H*_2_).

### Dynamic Governing Equations

**Figure 3:**
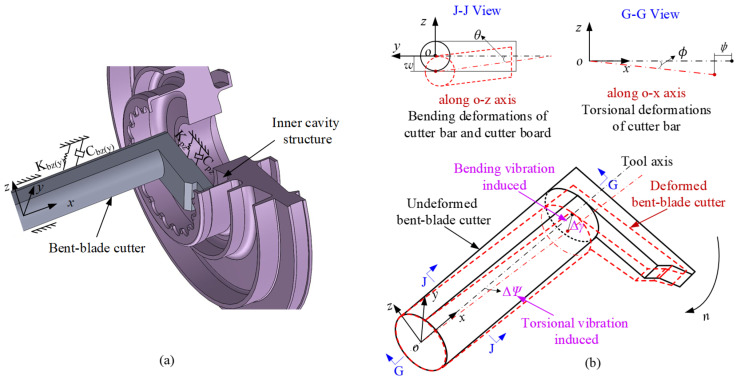
Dynamic model of the bent-blade cutter. (**a**) Turning process of the cutter; (**b**) Dynamic model considering the combined effect of bending and torsion.

In this section, the governing equations of the cutter bar beam and the cutter board beam are presented respectively. Then the dynamic model of the bent-blade cutter is established. The bent-blade cutter can be considered to have three orthogonal degrees of freedom, as shown in Figure 3a.

Figure 3 illustrates the bent-blade cutter during the machining process. The cutter experiences cutting forces and torque, leading to bending and torsional vibrations. Due to these vibrations, the deformed bent-blade cutter shown by the dashed line deviates from the undeformed one shown by the solid line. That is, the cutter’s actual cutting trajectory deviates from the nominal cutting trajectory. To exactly describe the motion of the cutter bar beam and cutter board under the action of cutting force, four displacement components, namely *w*, *θ*, *ψ* and *ϕ* are needed. *w*, *θ*, *ψ* and *ϕ* represent the transverse displacement along the *o-z* axis, rotation angle displacement of the cross-section, the warping displacement along the *o-x* axis and twist angular displacement of the cross-section in the local coordinate system, respectively.

(1) $$\begin{array}{l} \begin{array}{ccc} \varepsilon_{xx}=-z\frac{\partial \phi }{\partial x}, & \varepsilon_{yy}=0, & \varepsilon_{zz}=0 \end{array}\\ \begin{array}{ccc} \varepsilon_{xy}=\left(\frac{\partial \psi }{\partial y}-z\right)\frac{\partial \theta }{\partial x}, & \varepsilon_{yz}=0 & \end{array}\\ \varepsilon_{zx}=-\phi \left(x,t\right)+\frac{\partial w}{\partial x}+\left(\frac{\partial \psi }{\partial z}+y\right)\frac{\partial \theta }{\partial x} \end{array}$$

The cutter bar beam is not only subjected to the shear deformations and rotary inertia, but also the torsional deformations. As shown in Figure 3b, *w*, *θ*, *ψ* and *ϕ* are considered simultaneously, and the strain–displacement relationship is shown as Equation (1).

The corresponding constitutive relationship is

(2) $$\begin{array}{l} \begin{array}{ccc} \sigma_{xx}=-Ez\frac{\partial \phi }{\partial x}, & \sigma_{yy}=0, & \sigma_{zz}=0 \end{array}\\ \begin{array}{ccc} \sigma_{xy}=k_{s}G\left(\frac{\partial \psi }{\partial y}-z\right)\frac{\partial \theta }{\partial x}, & \sigma_{yz}=0 & \end{array}\\ \sigma_{zx}=k_{s}G\left[\frac{\partial w}{\partial x}-\phi \left(x,t\right)+\left(\frac{\partial \psi }{\partial z}+y\right)\frac{\partial \theta }{\partial x}\right] \end{array}$$

where *σ_xx_*, *σ_yy_*, *σ_zz_* are normal stress, *σ_xy_*, *σ_yz_*, *σ_zx_* are shear stress. *G* and *E* represent the shear modulus and elasticity modulus of the bent-blade cutter; *k_s_* is the shear coefficient. Based on the E-TBM considering the shear deformations, the torsional deformations, the warping deformations and rotary inertia effects, combined with constitutive relationship Equations (1) and (2), the strain energy *U* is [32]

(3) $$\begin{array}{cc} U & =\int_{0}^{l}\left[EI\frac{\partial \phi }{\partial x}\delta \left(\frac{\partial \phi }{\partial x}\right)+k_{s}AG\left(\frac{\partial w}{\partial x}-\phi \right)\delta \left(\frac{\partial w}{\partial x}\right)-k_{s}AG\left(\frac{\partial w}{\partial x}-\phi \right)\delta \phi \right]dx\\ & +\int_{0}^{l}\iint_{A}k_{s}G\left\{\begin{array}{c} \left[{\left(\frac{\partial \psi }{\partial y}-z\right)}^{2}+{\left(\frac{\partial \psi }{\partial z}+y\right)}^{2}\right]\frac{\partial \theta }{\partial x}\delta \left(\frac{\partial \theta }{\partial x}\right)\\ +{\left(\frac{\partial \theta }{\partial x}\right)}^{2}\left[\left(\frac{\partial \psi }{\partial y}-z\right)\delta \left(\frac{\partial \psi }{\partial y}\right)+\left(\frac{\partial \psi }{\partial z}+y\right)\delta \left(\frac{\partial \psi }{\partial z}\right)\right] \end{array}\right\}dAdx\\ & +\int_{0}^{l}\iint_{A}k_{s}G\left[\begin{array}{l} \left(\frac{\partial \psi }{\partial z}+y\right)\left(\frac{\partial w}{\partial x}-\phi \right)\delta \left(\frac{\partial \theta }{\partial x}\right)+\frac{\partial \theta }{\partial x}\left(\frac{\partial w}{\partial x}-\phi \right)\delta \left(\frac{\partial \psi }{\partial z}\right)\\ +\frac{\partial \theta }{\partial x}\left(\frac{\partial \psi }{\partial z}+y\right)\delta \left(\frac{\partial w}{\partial x}\right)-\frac{\partial \theta }{\partial x}\left(\frac{\partial \psi }{\partial z}+y\right)\delta \phi \end{array}\right]dAdx \end{array}$$

The kinetic energy *T* can be given by

(4) $$T=\int_{0}^{l}\left(\rho A\frac{\partial^{2}w}{\partial t^{2}}\delta w+\rho I\frac{\partial^{2}\phi }{\partial t^{2}}\delta \phi +\rho I_{p}\frac{\partial^{2}\theta }{\partial t^{2}}\delta \theta \right)dx$$

where *ρ* is the density, *I* is the area moment of inertia of the cutter bar beam cross-section, *I*_p_ is the polar moment of inertia of the cutter bar beam cross-section, *A* is the area of cross-section.

Additionally, the work *W* done by cutting force *F* along *x*-axis and torque *J* is given by

(5) $$W=\int_{0}^{l}\left(Fw+J\theta \right)dx$$

(6) $$\begin{array}{c} -\frac{\partial }{\partial x}\left[k_{s}AG\frac{\partial }{\partial x}\left(\frac{\partial w}{\partial x}-\phi \right)\right]-\iint_{A}k_{s}G\left(\frac{\partial \psi }{\partial z}+y\right)dA\frac{\partial }{\partial x}\left(\frac{\partial \theta }{\partial x}\right)+\rho A\frac{\partial^{2}w}{\partial^{2}t}-F=0\\ -\frac{\partial }{\partial x}\left(EI\frac{\partial \phi }{\partial x}\right)-\iint_{A}k_{s}G\left(\frac{\partial \psi }{\partial z}+y\right)dA\frac{\partial \theta }{\partial x}-k_{s}AG\left(\frac{\partial w}{\partial x}-\phi \right)+\rho A\frac{\partial^{2}\phi }{\partial^{2}t}=0\\ -\frac{\partial }{\partial x}\left(k_{s}\frac{\partial \theta }{\partial x}\right)-\iint_{A}k_{s}G\left(\frac{\partial \psi }{\partial z}+y\right)dA\frac{\partial }{\partial x}\left(\frac{\partial w}{\partial x}-\phi \right)+\rho I_{p}\frac{\partial^{2}\theta }{\partial^{2}t}-J=0\\ \frac{\partial^{2}\psi }{\partial y^{2}}+\frac{\partial^{2}\psi }{\partial z^{2}}=0 \end{array}$$

Based on the Hamilton theory, the governing equation of cutter bar beam in the local coordinate system is obtained.

From Equation (6), the cutter bar beam model contains the shear deformations, torsional deformations, warping deformations and rotary inertia effects. Because the cross-section of the cutter bar is regarded as circular, the warping deformations of the cutter bar beam are neglected. For the convenience of study, it is assumed that the bending and torsion effects of the element are uncoupled. Therefore, the governing equations of the cutter bar beam can be written as

(7) $$\begin{array}{c} -\frac{\partial }{\partial x}\left[k_{s}AG\frac{\partial }{\partial x}\left(\frac{\partial w}{\partial x}+\phi \right)\right]+\rho A\frac{\partial^{2}w}{\partial^{2}t}-F=0\\ -\frac{\partial }{\partial x}\left(EI\frac{\partial \phi }{\partial x}\right)-k_{s}AG\left(\frac{\partial w}{\partial x}+\phi \right)+\rho A\frac{\partial^{2}\phi }{\partial^{2}t}=0\\ -\frac{\partial }{\partial x}\left(k_{s}GI_{p}\frac{\partial \theta }{\partial x}\right)+\rho I_{p}\frac{\partial^{2}\theta }{\partial^{2}t}-J=0 \end{array}$$

**Figure 4:**
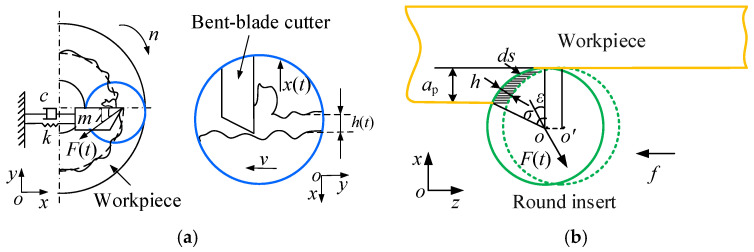
Modeling of regenerative vibration. (**a**) Self-excited vibration model of the cutter turning process; (**b**) Analysis of the dynamic chip thickness of cutter.

As shown in Figure 4b, the circular blade is different from the traditional cutting blade, its undeformed chip thickness is constantly changing along the cutting edge. Therefore, combining with the processing characteristics of the bent-blade cutter, the dynamic cutting force model of the circular blade can be obtained [33].

It is assumed that the cutting force *F*(*t*) is proportional to the instantaneous chip thickness *h* by coupling the movement of the tool and the parts. For a circular blade, chip thickness constantly changes along the cutting edge. Therefore, the cutting force *F*(*t*) of the macroscopic circular blade is discretized into differential component forces *dF*(*t*,*ε*) along the cutting edge, and the cutting force *dF*(*t*) is divided into as the following three parts according to the machining conditions.

(8) $$dF(t,\varepsilon )=k_{t}\left[\begin{array}{c} k_{n}\cos\varepsilon \\ 1\\ k_{n}\sin\varepsilon \end{array}\right]h(t,\varepsilon )ds$$

where *k_t_* is the tangential cutting force coefficient, *k_n_* is the ratio of the normal cutting force to the tangential cutting force and *ε* represents the angular location of the infinitesimal segment at the cutting edge. Assuming that the direction of the component force *dF*(*t*,*ε*) is parallel to the radial direction of the circular blade, the chip thickness *h*(*t*,*ε*) can be expressed as

(9) $$h(t,\varepsilon )=\left[\begin{array}{ccc} \cos\varepsilon & 0 & \sin\varepsilon \end{array}\right]\Delta l(t)$$

The vector Δ*l*(*t*) = [Δ*x*(*t*), Δ*y*(*t*), Δ*z*(*t*)]^T^ represents the relative vibration displacement between the tool and the workpiece.

In this paper, only the relative vibrations in the *x* and *z* directions are considered. Therefore, the relative vibration displacement in Equation (9) can be written as [34],

(10) $$\begin{array}{l} \Delta x(t)=\left(1-\frac{\arcsin\frac{f}{2r}}{\arccos\frac{r-a_{p}}{r}}\right)x(t-T)-x(t)\\ \Delta z(t)=z(t-T)-z(t)+fT \end{array}$$

where *f* is the cutter feed, *r* is the cutter radius. By substituting Equation (10) into Equation (9) and setting *ds* = *rdε*, the matrix of component force *dF*(*t*,*ε*) can be obtained as follows:

(11) $$dF(t,\varepsilon )=K_{t}r\left[\begin{array}{ccc} \cos\varepsilon & 0 & \sin\varepsilon \\ k_{n}\cos^{2}\varepsilon & 0 & k_{n}\sin\varepsilon \cos\varepsilon \\ k_{n}\sin\varepsilon \cos\varepsilon & 0 & k_{n}\sin^{2}\varepsilon \end{array}\right]\Delta l(t)d\varepsilon$$

Therefore, the cutting force *F*(*t*) can be expressed as

(12) $$F(t)=\int_{0}^{\delta }dF(t,\varepsilon )=K_{t}b\Omega (\delta )\Delta l(t)$$

with

(13) $$\cos\delta =1-\frac{b}{r}$$

(14) $$\Omega (\delta )=\left[\begin{array}{ccc} \delta_{xx} & 0 & \delta_{xz}\\ \delta_{yx} & 0 & \delta_{yz}\\ \delta_{zx} & 0 & \delta_{zz} \end{array}\right]$$

(15) $$\begin{array}{l} \begin{array}{ccc} \delta_{xx}=\frac{\sin\varepsilon }{1-\cos\varepsilon }, & \delta_{xz}=\delta_{zx}=1, & \delta_{yx}=k_{n}\frac{2\delta +\sin2\delta }{1-\cos\delta } \end{array},\\ \begin{array}{ccc} \delta_{yz}=k_{n}\frac{1-\cos2\delta }{4\left(1-\cos\delta \right)}, & \delta_{zz}=k_{n}\frac{2\delta -\sin2\delta }{4\left(1-\cos\delta \right)} & \end{array} \end{array}$$

where *b* is the cutting width. The relationship between the direction coefficient and the cutting width *b* is nonlinear. According to Equation (13), the engagement angle *δ* changes non-linearly with the change of cutting width *b*.

Because of the poor rigidity of the bent-blade cutter, it is easy to produce chatter in the inner cavity structure turning process. As the rigidity of deep inner cavity parts is larger than the cutter, the turning system model of the bent-blade cutter is established considering the vibration of the cutter in the *x* direction only, as shown in Figure 4a. According to the established model of the bent-blade cutter turning system in Figure 4a, the dynamic governing equation of the cutter can be expressed as follows:

(16) $$m\ddot{x}(t)+c\dot{x}(t)+kx(t)=F(t)\cos\varepsilon$$

where *m*, *c* and *k* represent the equivalent mass, equivalent damping and equivalent stiffness of the bent-blade cutter in the *x* direction, respectively. According to Equation (12), the component of *F*(*t*) in the *x* direction can be determined as follows:

(17) $$F(t)\cos\varepsilon =k_{t}b\left\{\delta_{xx}\left[\left(1-\frac{\arcsin\frac{f}{2r}}{\arccos\frac{r-a_{p}}{r}}\right)x(t-T)-x(t)\right]+\delta_{xz}\left(z(t-T)-z(t)\right)\right\}$$

## 3. Finite Element Analyses

The FEM is utilized to solve the governing equations of cutter bar beam. A two-node Timoshenko beam element is developed. Three degrees of freedom are assigned to each node, i.e., shear deformations, torsion angle and rotation angle of cross-section. According to the variation principle [34], Equation (6) can be expressed as

(18) $$\begin{array}{l} 0=\int_{x_{a}}^{x_{b}}\left[k_{s}GA\frac{dv_{1}}{dx}\left(\frac{\partial w}{\partial x}+\phi \right)+\rho Av_{1}\frac{\partial^{2}}{\partial^{2}t}-v_{1}F\right]dx-v_{1}\left(x_{a}\right)Q_{1}^{e}-v_{1}\left(x_{b}\right)Q_{3}^{e}\\ 0=\int_{x_{a}}^{x_{b}}\left[EI\frac{dv_{2}}{dx}\frac{\partial \phi }{\partial x}-v_{2}k_{s}AGv_{2}\left(\frac{\partial w}{\partial x}+\phi \right)+\rho Av_{2}\frac{\partial^{2}\phi }{\partial^{2}t}\right]dx-v_{2}\left(x_{a}\right)Q_{2}^{e}-v_{2}\left(x_{b}\right)Q_{4}^{e}\\ 0=\int_{x_{a}}^{x_{b}}\left[k_{s}GI_{p}\frac{dv_{3}}{dx}\frac{\partial \theta }{\partial x}+\rho I_{p}v_{3}\frac{\partial^{2}\theta }{\partial^{2}t}-v_{3}J\right]dx-v_{3}\left(x_{a}\right)Q_{5}^{e}-v_{3}\left(x_{b}\right)Q_{6}^{e} \end{array}$$

where *Q_i_^e^* (*i* = 1, 2, …, 6) are shown as follows:

(19) $$\begin{array}{l} Q_{1}^{e}=-{\left[k_{s}AG\left(\phi +\frac{dw}{dx}\right)\right]}_{x=x_{a}}=-V\left(x_{a}\right)\\ Q_{2}^{e}=-{\left(EI\frac{d\phi }{dx}\right)}_{x=x_{a}}=-M\left(x_{a}\right)\\ Q_{3}^{e}={\left[k_{s}AG\left(\phi +\frac{dw}{dx}\right)\right]}_{x=x_{b}}=V\left(x_{b}\right)\\ Q_{4}^{e}={\left(EI\frac{d\phi }{dx}\right)}_{x=x_{b}}=-M\left(x_{b}\right)\\ Q_{5}^{e}={\left(k_{s}GI_{p}\frac{d\theta }{dx}\right)}_{x=x_{a}}=T\left(x_{a}\right)\\ Q_{6}^{e}=-{\left(k_{s}GI_{p}\frac{d\theta }{dx}\right)}_{x=x_{b}}=-T\left(x_{b}\right) \end{array}$$

For the cutter board beam, it should be noted that the twist angle of the cross-section is zero because of the absence of torsional deformation under the action of the cutting force. The governing equations are obtained directly based on the Timoshenko theory in the local coordinate system, which are shown in the first two of Equation (9). According to the variation principle, the final weak form of Equation (6) over an element (*x_a_*, *x_b_*) can be expressed as shown in the first two of Equation (18).

After deriving the cutter bar beam and the cutter board beam element stiffness matrix and mass matrix, respectively, the crucial task is to connect the two beams to ensure tool continuity. Subsequently, the element stiffness matrices and element mass matrices of the cutter bar beam and the cutter board beam needed to be transformed from local coordinates to global coordinates. Finally, the motion equations for the bent-blade cutter were established, and analytical methods were employed to obtain the static deformation and dynamic characteristics of the tool. For detailed derivation steps, please refer to reference [32].

The DQM is used to obtain the stability lobe diagram of the cutter. The idea of the DQM method is that the partial derivative of the function relative to the coordinate direction of grid points sampled within the interval along this direction can be approximately the linear weighted sum of all function values of grid points sampled within the entire interval. In this paper, the dynamic Equation (19) of the bent-blade cutter is approximated to a set of algebraic equations by the DQM, and then Floquet transformation matrix is constructed to analyze the turning stability [31]. Compared with other stability prediction methods, the semi-analytical time-domain method is simpler and more efficient.

## 4. Simulation and Experimental Validations

### 4.1. Setup of the Experiment

**Figure 5:**
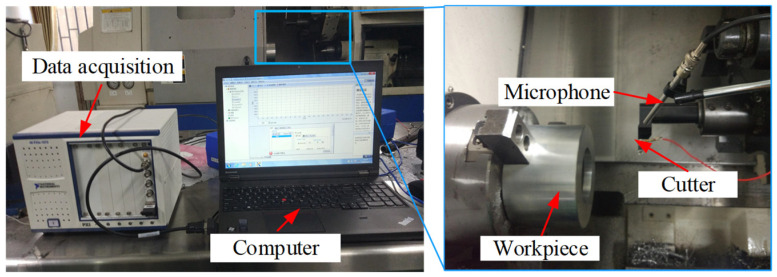
Setup in turning tests.

In order to verify the correctness of the prediction model proposed in this paper, a series of standard turning tests and impact tests are conducted on the bent-blade cutter. As shown in Figure 5, the turning tests are carried out in the CNC lathe center (14.9 kW, 4500 rpm). A GRAS 40 pp microphone with a sensitivity of 50 mV/Pa is used to collect the turning sound signals (Holte, Denmark). The tool bar of the bent-blade cutter is fixed on the tool holder, and the overhanging length is 110 mm × 60 mm, which is the same as that of the impact tests. The round blade material is hard alloy, and the blade radius *r* is 4 mm.

The geometric parameters of the bent-blade cutter are shown in Table 1. The cross-section of cutter is circular and the cross-section shear coefficient *k_s_* is 0.9. The material of the cutter is mild steel, Young’s modulus of elasticity *E* = 211 GPa, density *ρ* = 7800 kg/m^3^ and Poisson’s ratio *μ* = 0.3. In order to verify the machining stability of the bent-blade cutter, the workpiece with larger diameter is selected to reduce the vibration of the workpiece in the cutting experiment and obtain more reliable verification results. The material of the workpiece is aluminum alloy 7075. Its wall thickness, outside diameter and length are 20 mm, 200 mm and 250 mm, respectively. Compared with thin-walled parts with 1–2 mm wall thickness, the deep inner workpiece with larger diameter is a rigid body. Therefore, only the weak rigidity of the cutter is considered in this paper.

**Figure 6:**
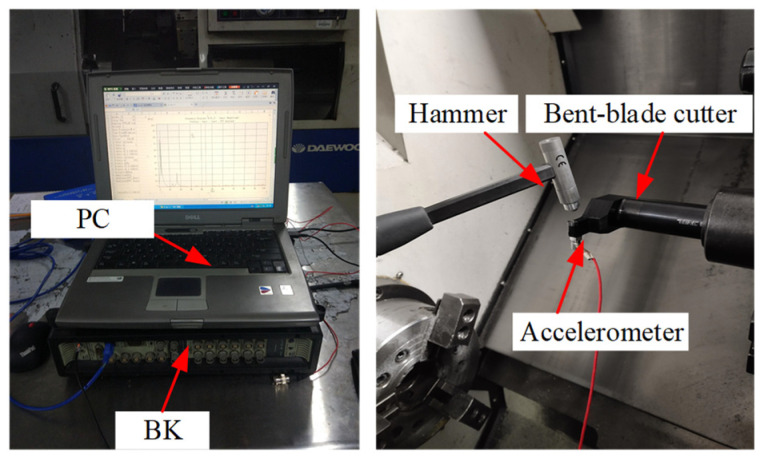
Setup in dynamic tests.

**Figure 7:**
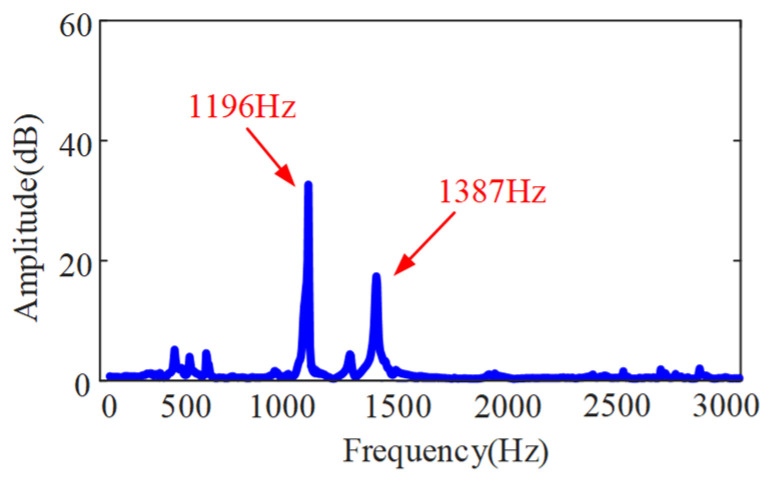
FRFs of the bent-blade cutter.

As shown in Figure 6, the impact tests are carried out on the bent-blade cutter. The vibration signal of the tool under the excitation force is measured by an accelerometer with a sensitivity of 98.45 mV/g. Meanwhile, the signal collected is recorded by a B&K data acquisition system. A force hammer (CL-YD-303, Sinocera Piezotronics Inc., Saint Lucia, Australia) with a sensitivity of 4.29 Pc/N is used to exert the hammering force on the bending tool. The obtained frequency response function is shown in Figure 7.

### 4.2. Chatter Tests and Results Analysis

**Figure 8:**
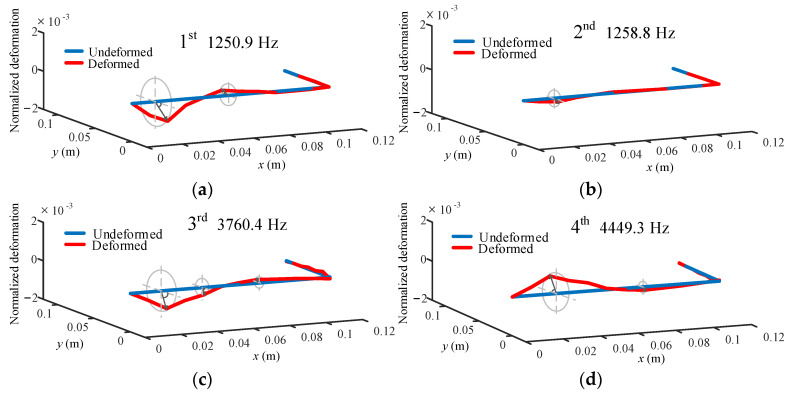
First four modes of vibration evaluated by the present method. (**a**) First order mode; (**b**) Second order mode; (**c**) Third order mode; (**d**) Fourth order mode.

In the turning process, the modal characteristics of the bent-blade cutter have a significant effect on the turning stability of the deep inner cavity. According to the dynamic model of the bent-blade cutter established in Section 2 and the geometric parameters and material properties of cutter in Table 1, the modal parameters are obtained. Correspondingly, the simulated first four mode shapes and the maximum deformation position of each mode shape of the cutter are given in Figure 8. Among them, the blue solid line represents the first four order modes of the bent-blade cutter without considering the bending and torsional deformation effects, and the red solid line represents the first four modes with considering the bending and torsional deformation effects. From Figure 8, it can be seen that:

1. The first four order natural frequencies of the bent-blade cutter considering the combined deformation effect of bending and torsion are 1250.9 Hz, 1258.8 Hz, 3760.5 Hz and 4449.3 Hz. The first two modes have the modal agglomeration phenomenon, which is caused by the structure symmetry of the simplified dynamical model of the bent-blade cutter.
2. The bending and torsional deformation of the tool mainly occur on the cutter bar, and the bending deformation at the cutter bar is larger than the torsional deformation.
3. Because the cutter board is connected to the place where the cutter bar has the greatest torsion, the torsional vibration mode at the cutter board is larger than the bending vibration mode.

In addition, it can be seen from Figure 7 that the first two order natural frequencies of the cutter obtained through impact tests are 1196 Hz and 1387 Hz, respectively. They are in good agreement with the results calculated by the method in this paper, and the error is 9%. The above phenomena prove that the influence of torsional and bending angular displacements on the vibration of the bent-blade cutter cannot be ignored in the deep inner cavity part turning process, and are needed for predicting the SLDs.

**Figure 9:**
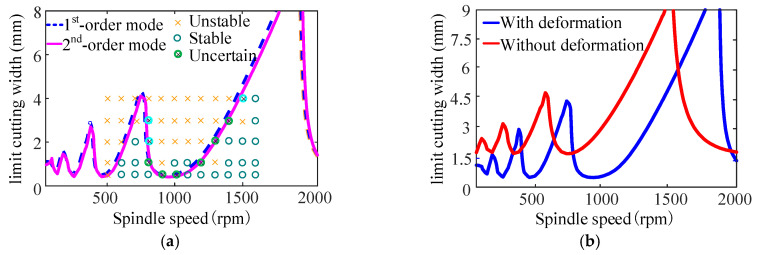
SLD of bent-blade cutter; (**a**) The SLD of bent-blade cutter with the first two order modal parameters; (**b**) The SLD of bent-blade cutter with bending and torsion deformation.

**Figure 10:**
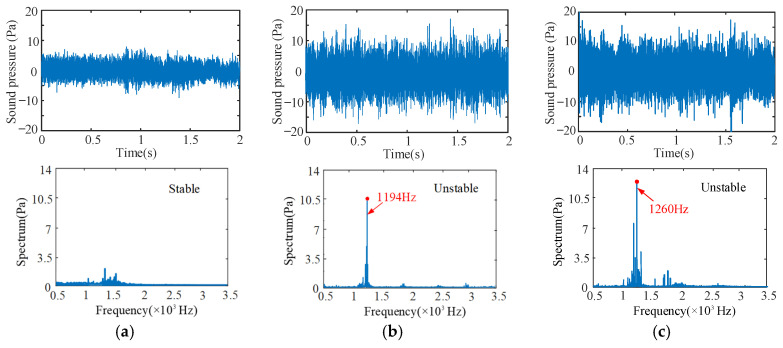

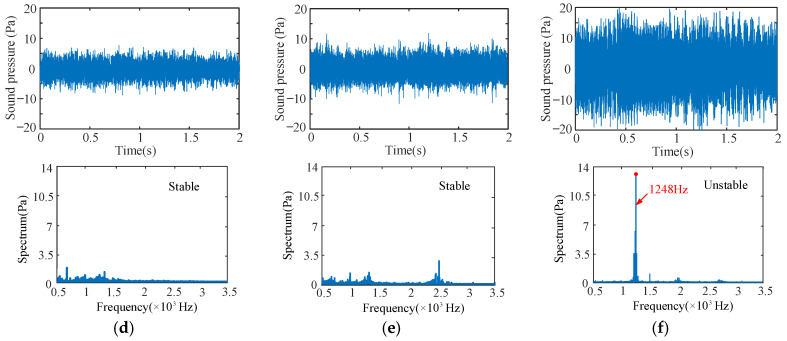
Time-frequency domain sound signals measured in experiments ((**a**) *n* = 600 rpm, *b* = 0.5 mm; (**b**) *n* = 600 rpm, *b* = 2 mm; (**c**) *n* = 700 rpm, *b* = 3 mm; (**d**) *n* = 1400 rpm, *b* = 2 mm; (**e**) *n* = 1400 rpm, *b* = 3 mm; (**f**) *n* = 1400 rpm, *b* = 4 mm).

To validate the model proposed in Section 2, the differential quadrature method was employed to predict the SLDs for the bent-blade cutter turning process, as illustrated in Figure 9. The modal parameters are detailed in Table 2. The first two modal parameters of the bent-blade cutter are studied, and the influence of the mode-coupling effect on the turning stability prediction is discussed. Figure 9a shows the SLD of the bent-blade cutter, the blue dotted line represents the SLD obtained by the first-order modal parameters and the purple solid line represents the SLD obtained by the second-order modal parameters. The influence of the combined bending and torsion deformation effect of the bent-blade cutter on the turning stability is discussed through the obtained SLD as shown in Figure 9b. The predicted and experimentally observed stability results for the turning of the bent-blade cutter are shown in Figure 9a, respectively. Cutting conditions under the critical cutting width of cut are chatter-free; otherwise, they will be in an unstable state. The spindle speed is changed from 500 to 1600 rpm with an increment of 100 rpm. The limit cutting width of the cut is changed from 0.5 to 4 mm with increments of 0.5 mm and 1 mm. Sixty cutting conditions are used for turning of the bent-blade cutter. The experimental results are in good agreement with those predicted. Samples of the measured sound signals are presented at different cutting conditions in Figure 10.

From Figure 9a, it can be seen that there is a large modal coupling effect in the stable lobes obtained according to the first two order modal parameters of the bent-blade cutter. It is different from the milling of thin-walled parts; the mode of the bent-blade cutter remains unchanged. Therefore, the bent-blade cutter also has multi-modal characteristics. From Figure 9a, although a few test points are contrary to the predicted results, including the critical cutting states that are difficult to determine, the experimental results are generally in good agreement with the predicted results. This may be caused by the inevitable influencing factors, such as the interference of vibration signals caused by the bending, torsion frequency, chatter frequency and workpiece burr in the actual machining process. In addition, the setting of the cutting force coefficient has an inevitable impact on the results in the model establishment.

As shown in Figure 9b, the effect of combined bending and torsion deformation on the turning stability of bent-blade cutters is studied using the SLD obtained by the first-order modal parameters. The blue solid line represents the SLD considering the combined bending and torsion deformation effect, and the red solid line represents the SLD without the combined bending and torsion deformation effect. When the effect of combined bending and torsion deformation on the turning stability of the bent-blade cutter is not considered, the stable lobes move up and left. It is obviously different from the stable lobes considering the combined bending and torsion deformation effect. The reason is that the bent-blade cutter is more prone to vibration in the turning process after considering the combined bending and torsion deformation effect, which reduces the machining stability. Therefore, it can be concluded that the combined deformation effect of bending and torsion of the bent-blade cutter in the deep inner cavity part turning process cannot be ignored.

**Figure 11:**
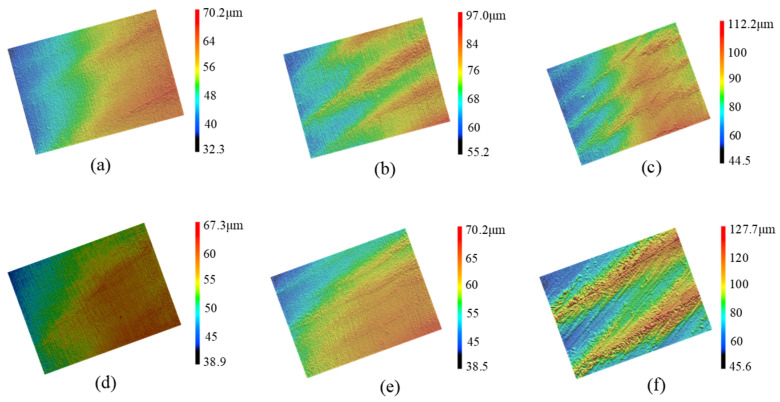
Surface morphology. ((**a**) *n* = 600 rpm, *b* = 0.5 mm; (**b**) *n* = 600 rpm, *b* = 2 mm; (**c**) *n* = 700 rpm, *b* = 3 mm; (**d**) *n* = 1400 rpm, *b* = 2 mm; (**e**) *n* = 1400 rpm, *b* = 3 mm; (**f**) *n* = 1400 rpm, *b* = 4 mm).

Figure 10 shows the samples of the measured sound pressure signals under different cutting conditions. The spindle speed is 600 rpm and the limit cutting widths are 0.5 mm and 2 mm in Figure 10a,b. The spindle speed is 700 rpm and the limit cutting widths is 3 mm in Figure 11c. The spindle speed is 1400 rpm and the limit cutting widths are 2 mm, 3 mm and 4 mm in Figure 11d–f. The time domain and frequency domain signals of the sound pressure signals are analyzed.

Due to the low dynamic stiffness of the overhanging part of the bent-blade cutter, the chatter frequency appears near the first natural frequency of 1250.9 Hz. Therefore, its stability is mainly determined by the first mode parameter. As can be seen from the time-frequency domain analysis in Figure 10b,c,f, the vibration signals fluctuate greatly, and the vibration frequencies are all near the natural frequencies, with obvious chatter. Furthermore, it can also be seen from the machined surface topography in Figure 11b,c,f that there are obvious vibration patterns on the surface, and the amplitude peak value of vibration patterns increases gradually along the direction of the cutting path, which are agreement with the results of Figure 10.

The cutting process corresponding to the cutting additions of Figure 10a,d,e under the critical cutting width of cut is in a stable state. Similarly, as can be seen from Figure 11a,d,e, there are no vibration marks on the machined surface, and the surface integrity is good. These results are agreement with the predicted results. In addition, when the spindle speed is 1400 rpm, the cutting chatter is more likely to occur with the increase in cutting width, as shown in Figure 10. A similar phenomenon can also be obtained in other different cutting conditions.

### 4.3. Influence of Cutting Parameters on Turning Stability

Based on the research results of bent-blade cutter turning stability in Section 4.2, the turning stability of the bent-blade cutter is not only related to the natural frequency, modal mass, modal stiffness, damping ratio and cutting force coefficient of the cutter, but also related to the turning parameters, such as spindle speed, feed and cutting width. From the stability lobes, it is directly analyzed that the corresponding limit cutting width is different at different spindle speeds, which has an impact on the turning stability of the bent-blade cutter. The feed and cutting width affect the stability of the cutter by affecting the cutting force. For the turning process, cutting parameters directly affect the stability of turning. Therefore, it is necessary to carry out quantitative analysis of cutting parameters. The detailed analysis is as follows.

#### 4.3.1. The Effect of Spindle Speed on Turning Stability

As shown in Figure 9a, the limit cutting width changes periodically with the increase in spindle speed. The stability lobe diagram of the first two modal parameters of the cutter shows a similar trend. When the spindle speed is 400 rpm at the lowest point of the SLD, the minimum value of the limit cutting width is 0.40 mm. When the spindle speed is various from 950 rpm to 1050 rpm, the minimum value of the limit cutting width is 0.38 mm. And when the spindle speed is 700 rpm, the limit cutting width is 3.19 mm, about 8 times the minimum value of the limit cutting width. When the spindle speed is 1800 rpm, the limit cutting width is 8.95 mm, about 24 times the minimum value.

It is obvious from Figure 9a that when the spindle speed is in the cutting range of 850~1250 rpm, the chatter of the bent-blade cutter can be avoided by increasing the spindle speed. When the spindle speed is in the cutting range of 400~600 rpm, the bent-blade cutter has chatter. Therefore, reducing the spindle speed is an effective way to avoid chatter. On the premise of ensuring the machining quality of the workpiece, selecting an optimal spindle speed can not only improve the machining efficiency but also avoid the chatter phenomenon.

#### 4.3.2. The Effect of Feed on Turning Stability

**Figure 12:**
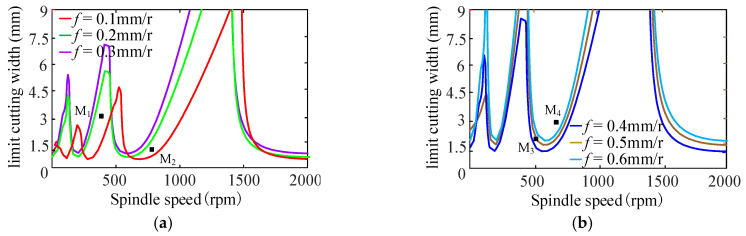
Effect of feed on stability lobe diagram. (**a**) *f* = 0.1~0.3 mm/r; (**b**) *f* = 0.4~0.6 mm/r.

Figure 12 shows the SLD of the feed effect on the turning process when the feed ranges from 0.1 mm/r to 0.6 mm/r. Figure 12a represents the stability lobe diagram corresponding to the feed *f* = 0.1~0.3 mm/r, and Figure 12b represents the stability lobe diagram corresponding to the feed *f* = 0.4~0.6 mm/r.

**Figure 13:**
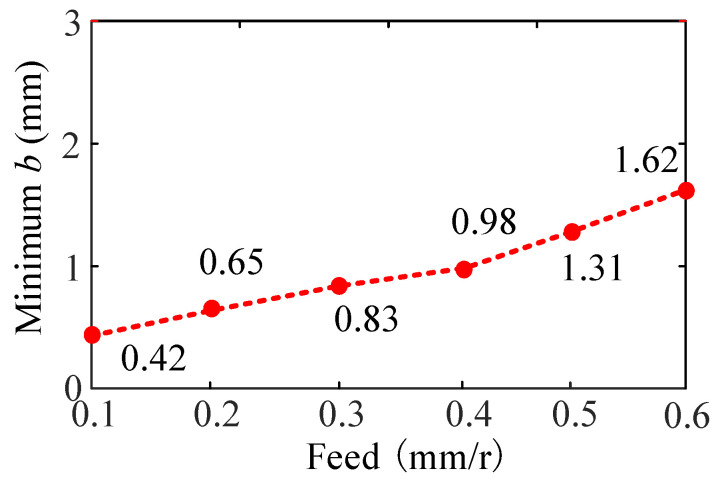
Effect of feed on the minimum cutting width.

From Figure 12, when other cutting parameters are the same, the stable lobe diagram shifts to left in the direction parallel to the spindle speed with the increase in feed. At the same time, the stable lobe diagram shifts upward, and the minimum value of the limit cutting width increases with the increase in feed, and then, the turning stability of the bent-blade cutter increases, as shown in Figure 13. The reason is that the increase in the feed reduces the overlap coefficient of the two cutting processes and makes the cutting process more stable.

**Figure 14:**
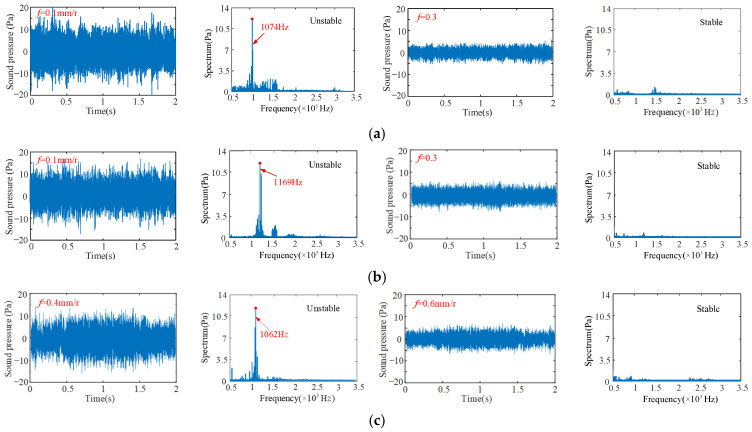

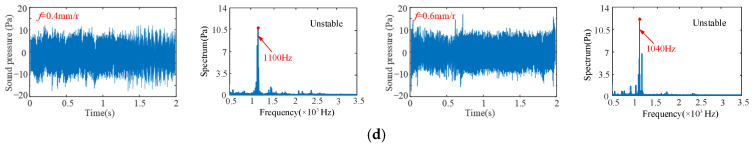
The sound signals in the time domain and the FFT of the cutter. (**a**) at point M_1_; (**b**) at point M_2_; (**c**) at point M_3_; (**d**) at point M_4_, for different feeds.

**Figure 15:**
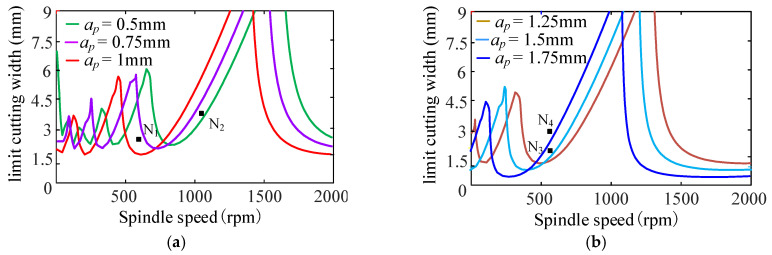
Effect of cutting depth on stability lobe diagram. (**a**) *a_p_* = 0.5~1 mm; (**b**) *a_p_* = 1.25~1.75 mm.

To validate the prediction results of feed on turning stability, four different feeds are selected respectively, i.e., 0.1 mm/r, 0.3 mm/r, 0.4 mm/r and 0.6 mm/r at four different cutting points M_1_ (*n* = 400 rpm, *b* = 3 mm), M_2_ (*n* = 850 rpm, *b* = 1.5 mm), M_3_ (*n* = 500 rpm, *b* = 2 mm) and M_4_ (*n* = 700 rpm, *b* = 3 mm), as shown in Figure 13. The sound signals are illustrated in Figure 14, and the frequency spectra of the sound signals are given on the right side of each sound signal in the same figure. From Figure 15, it can be seen that there are obvious chatter waves at points M_1_ and M_2_ when *f* = 0.1 mm/r. When *f* = 0.3 mm/r, it is a stable state. Similarly, at point M_3_, it is a chatter state when *f* = 0.4 mm/r. When *f* = 0.6 mm/r, it is a stable state. At point M_4_, it is a chatter state when the feeds are 0.4 mm/r and 0.6 mm/r. Furthermore, the chatter frequency (1100 Hz) when *f* = 0.4 mm/r is closer to the tool natural frequency (1250.9 Hz), so the chatter is larger. When *f* = 0.6 mm/r, the chatter frequency is 1040 Hz, and the chatter is relatively small. Therefore, from the overall trend of change, it can be seen that, with the increase in feed, the cutting state gradually tends to a stable state. This is more obvious in Figure 13. In other words, at the same cutting position, with the increase in feed, the cutting state becomes more stable. The above analysis shows that the experimental results are the same as the predicted results.

#### 4.3.3. The Effect of Cutting Depth on Turning Stability

Figure 15 shows the SLD of the cutting width effect on the turning stability when the cutting width ranges from 0.5 mm to 1.75 mm. Figure 15a represents the stability lobe diagram corresponding to cutting depth *a_p_* = 0.5~1 mm. Figure 15b represents the stability lobe diagram corresponding to cutting depth *a_p_* = 1.25~1.75 mm.

**Figure 16:**
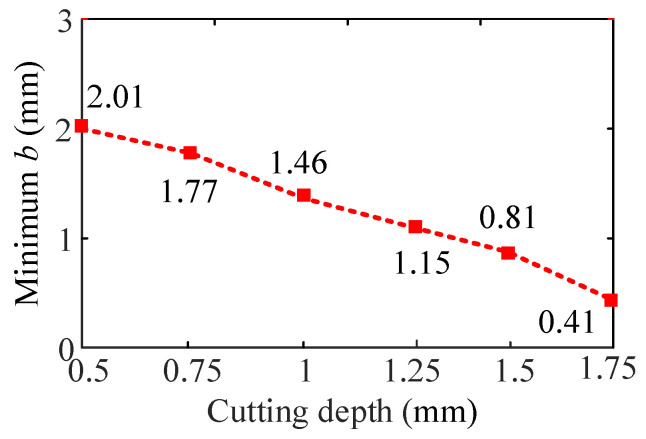
Effect of cutting depth on the minimum cutting width.

From Figure 15, it can be found that when other cutting parameters are the same, with the increase in cutting depth, the stable lobe diagram shifts to the left and down in the direction parallel to the spindle speed, and the stable region decreases. The minimum value of the limit cutting width decreases with the increase in cutting depth, as shown in Figure 16. Therefore, a smaller cutting depth can be used to reduce the turning vibration in the cutting process of the bent-blade cutter.

**Figure 17:**
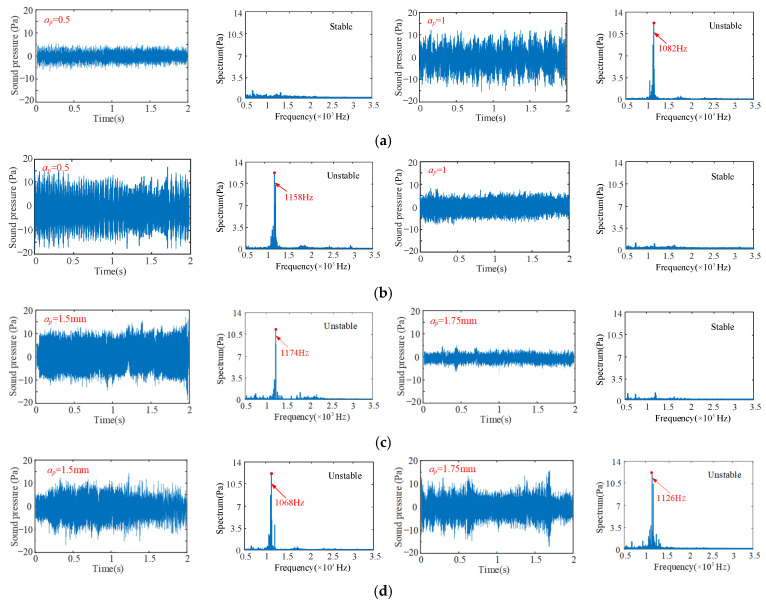
The sound signals in the time domain and the FFT of the cutter (**a**) at point N_1_; (**b**) at point N_2_; (**c**) at point N_3_; (**d**) at point N_4_, for different cutting depths.

Without loss of generality, four different cutting depths are selected to validate the predicted results of cutting depth on turning stability, respectively, i.e., *a_p_* = 0.5 mm, *a_p_* = 1 mm, *a_p_* = 1.5 mm and *a_p_* = 1.75 mm at four different cutting points N_1_ (*n* = 600 rpm, *b* = 2 mm), N_2_ (*n* = 1100 rpm, *b* = 3 mm), N_3_ (*n* = 600 rpm, *b* = 2 mm) and N_4_ (*n* = 600 rpm, *b* = 3 mm), as shown in Figure 16. The sound signals are illustrated in Figure 17, and the frequency spectra of the sound signals are given on the right side of each sound signal in the same figure. From Figure 17, it can be seen that at point N_1_, when *a_p_* = 0.5 mm, it is a stable state. When *a_p_* = 1 mm, there are obvious chatter waves. At point N_2_, when *a_p_* = 0.5 mm, it is a chatter state. When *a_p_* = 1 mm, it is stable. Similarly, at point N_3_, when *a_p_* = 1.5 mm, it is a chatter state. When *a_p_* = 1.75 mm, it is stable. However, the cutting depths 1.5 mm and 1.75 mm are both chatter states at point N_4_. In other words, at the same cutting position, the minimum value of the limit cutting width decreases with the increase in cutting depth. The above analysis shows that the experimental results are in good agreement with the predicted results. 

By studying the influence of spindle speed, feed and cutting depth on the turning stability of the bent-blade cutter, the cutting parameters in the turning process can be optimized and the stability of the bent-blade cutter can be improved. The results indicate that, under certain cutting conditions, increasing or decreasing the spindle speed can both help avoid chatter in the bent-blade cutter. Therefore, by selecting the optimal spindle speed while ensuring the quality of the workpiece, not only can machining efficiency be improved, but tool chatter can also be prevented. When other parameters remain unchanged, it is found that reducing the cutting depth and increasing the feed can effectively increase the stability region and improve the turning stability of the cutter. Therefore, the chatter can be effectively reduced by selecting an appropriate spindle speed, reducing the cutting depth and increasing the feed.

## 5. Conclusions

In order to predict the turning stability of the bent-blade cutter, this paper proposed a cutting stability prediction model considering the complex tool structure, turning characteristics of deep inner cavity parts, and the combined bending and torsion deformation effect. Though analyzing the stable lobes predicted and comparing the theoretical results with the experimental results, the following conclusions can be obtained.

1. There is a great modal coupling effect in the stable lobe diagram from the first two order modal parameters of the bent-blade cutter, but the mode of the cutter remains unchanged. Therefore, the cutter also has multi-modal characteristics: there are a modal coupling effect, and modal invariant characteristics.
2. Considering the influence of bending deformation and torsional deformation on the turning process, it can be seen from the analysis results that the stability lobes move up and left, and the turning stability is significantly improved. Therefore, the combined effect of bending and torsion cannot be ignored.
3. After determining the cutting system, an optimal spindle speed, cutting depth and a higher feed rate are selected in combination with the dynamic parameters to increase the stable area.

According to this study, the large overhang length of the cutter board and cutter bar of the bent-blade cutter in the deep inner cavity part turning process may lead to an unstable process. Therefore, the cutter manufacturer should consider the dynamic problem when designing the bent-blade cutter.

## Figures and Tables

**Table 1:** Geometry of the bent-blade cutter (unit: mm).

*L*_1_	*L*_2_	*L*_3_	*B*_1_	*B*_2_	*B*_3_	*H*_1_	*H*_2_	*D*
170	8	20	75	55	20	28	15	30

**Table 2:** First two modal parameters of the bent-blade cutter.

Modal Number	Natural Frequency (Hz)	Damping Ratio (×10^−2^)	The Modal Mass (kg)	Modal Stiffness (×10^7^ N/m)	Modal Damping (N·s/m)
1	1250.9	1.51	26.30	4.12	496.73
2	1258.8	1.46	25.70	4.07	472.40

## Data Availability

No new data were created or analyzed in this study. Data sharing is not applicable to this article.
